# Measurement of Adherence to mHealth Physical Activity Interventions and Exploration of the Factors That Affect the Adherence: Scoping Review and Proposed Framework

**DOI:** 10.2196/30817

**Published:** 2022-06-08

**Authors:** Yang Yang, Elisabeth Boulton, Chris Todd

**Affiliations:** 1 School of Health Sciences, Faculty of Biology, Medicine & Health University of Manchester Manchester United Kingdom; 2 Manchester Academic Health Science Centre Manchester United Kingdom; 3 Manchester University NHS Foundation Trust Manchester United Kingdom

**Keywords:** mobile health, mHealth, physical activity, adherence, framework, scoping review, mobile phone

## Abstract

**Background:**

Mobile health (mHealth) is widely used as an innovative approach to delivering physical activity (PA) programs. Users’ adherence to mHealth programs is important to ensure the effectiveness of mHealth-based programs.

**Objective:**

Our primary aim was to review the literature on the methods used to assess adherence, factors that could affect users’ adherence, and the investigation of the association between adherence and health outcomes. Our secondary aim was to develop a framework to understand the role of adherence in influencing the effectiveness of mHealth PA programs.

**Methods:**

MEDLINE, PsycINFO, EMBASE, and CINAHL databases were searched to identify studies that evaluated the use of mHealth to promote PA in adults aged ≥18 years. We used critical interpretive synthesis methods to summarize the data collected.

**Results:**

In total, 54 papers were included in this review. We identified 31 specific adherence measurement methods, which were summarized into 8 indicators; these indicators were mapped to 4 dimensions: length, breadth, depth, and interaction. Users’ characteristics (5 factors), technology-related factors (12 factors), and contextual factors (1 factor) were reported to have impacts on adherence. The included studies reveal that adherence is significantly associated with intervention outcomes, including health behaviors, psychological indicators, and clinical indicators. A framework was developed based on these review findings.

**Conclusions:**

This study developed an adherence framework linking together the adherence predictors, comprehensive adherence assessment, and clinical effectiveness. This framework could provide evidence for measuring adherence comprehensively and guide further studies on adherence to mHealth-based PA interventions. Future research should validate the utility of this proposed framework.

## Introduction

### Background

There is strong evidence that physical activity (PA) is associated with improvements in physical health, mental health, and well-being [1]. However, many people are at risk of inactivity and there is poor uptake of, and adherence to, PA interventions [2]. Promoting PA and maintaining people’s adherence are crucial public health issues.

Technological innovation (eg, mobile health [mHealth]) is developing rapidly and is being widely applied in the health care field [3]. mHealth mainly focuses on the delivery and monitoring of health care services [4] and could also be an alternative approach to delivering PA interventions, overcoming the limitations of traditional PA approaches (eg, classes or workshops) [5]. Compared with traditional approaches, mHealth can use vivid video and pictures and may be more attractive and acceptable [6]. The use of mHealth can help deliver exercise programs to a wide audience at a low cost. In addition, mHealth technologies can provide timely feedback, reminders and support, continuous monitoring, and outcomes assessment [7,8].

There is a common issue with innovative health technologies, including mHealth, which is users’ adherence to mHealth programs. For example, people who download an exercise app do not always use, or continue to use, the app. Research suggests that suboptimal exposure to the PA program lessens the effects of these interventions [9]. Measuring users’ adherence to mHealth and exploring the factors that could influence users’ adherence and the association between adherence and intervention outcomes are important to understand how PA and other outcomes can be improved.

Although there are systematic reviews summarizing evidence on the adherence to technology-based interventions, previous reviews are rather generic, do not differentiate mHealth from other technology-based interventions, do not focus on PA, and do not address the issue that adherence to a PA intervention may differ from adherence to other interventions such as those for medications or therapy. For example, Donkin et al [10] summarized measurement methods for adherence to any e-therapies and evaluated the association of adherence with intervention outcomes. Perski et al [11], focusing on digital behavior change interventions, developed a conceptual framework to explain the impacts of potential factors on people’s engagement with digital behavior change interventions. By contrast, other reviews may focus on PA but restrict themselves to specific digital technologies. For example, Attig et al [12], focusing on wearable trackers for PA, summarized reported reasons for abandoning their use, such as usability issues and privacy concerns.

mHealth is a commonly used innovative solution to deliver health interventions. The characteristics of instant access, portability, and direct feedback make mHealth different from other technologies such as desktop computers [4]. We believe that these characteristics could affect the adherence of users of mHealth-based interventions in a way that is different from adherence to other technologies. Therefore, it would be better to consider mHealth-based interventions more specifically rather than grouping them together with other, generic technologies. Given this, we consider that the measurement of users’ adherence to mHealth-based PA should not only reflect generic technologies’ features, where applicable, but also incorporate mHealth-specific factors. For example, the automatically recorded number of days when mHealth devices are worn and the automatically recorded daily amount of PA, such as step counts, are commonly used indicators in measuring users’ adherence to mHealth-based PA interventions [8]. However, such indicators have not been well considered in existing reviews that cover information technologies in general. Overall, there is a lack of evidence on determinants of the adherence to mHealth-based PA and the influence of adherence on health outcomes; in addition, there seems to be no agreed measurement method of adherence to mHealth devices that aim to improve PA engagement.

A scoping review of the literature was therefore carried out to explore how adherence to mHealth aiming at improving PA engagement is measured, to investigate which factors affect users’ adherence, and the association between adherence and intervention outcomes. A framework is needed to identify the association among factors, adherence measurement, and health outcomes. The framework can be used in future to guide the measurement of adherence to mHealth-based PA programs and facilitate further research on the effectiveness of mHealth-based programs.

### Objectives

The aims of this study were to synthesize evidence about (1) how adherence to mHealth PA interventions has been measured in the literature, (2) the factors that influence the adherence, and (3) the association between adherence to mHealth PA interventions and health outcomes. An additional aim was to propose an operational concept of adherence to mHealth-based PA programs and a framework for identifying the links among the determinants of adherence, adherence measurements, and intervention outcomes.

## Methods

The PRISMA-ScR (Preferred Reporting Items for Systematic Reviews and Meta-Analyses extension for Scoping Reviews) guidelines were followed for reporting this review [13]. Refer to Multimedia Appendix 1 for the PRISMA-ScR checklist. The review protocol was not registered on the web.

### Literature Search and Search Strategy

To identify relevant studies, the initial electronic searches were run in September 2020 in 4 databases: MEDLINE, PsycINFO, EMBASE, and CINAHL [14]. Update searches were performed in February 2022. Refer to Multimedia Appendix 2 for the search strategies. There were no restrictions on publication year, but searches were limited to English language publications.

### Eligibility Criteria

We considered a study eligible if it met the criteria presented in Table 1. Following the World Health Organization’s definitions of mHealth [15], we defined mHealth-based PA programs as interventions that use mobile devices to deliver PA. The devices could be smartphones, smartwatches, PDAs such as wristbands, and other wireless technologies. In addition, there were no restrictions on how adherence to mHealth interventions was defined and assessed in this review as long as the authors described the measurement or definition of adherence. Furthermore, this review used the PA definition provided by the World Health Organization [16]: any bodily movement produced by skeletal muscles that requires energy expenditure.

**Table 1:** Inclusion and exclusion criteria.

Items	Inclusion criteria	Exclusion criteria
Types of study	Any experimental and nonexperimental study design	Unpublished studies Papers that were not peer reviewed
Types of participants	People aged ≥18 years (including older adults)	Studies recruiting children (aged <18 years) or participants with cognitive impairment or psychiatric disorders
Types of interventions	Studies that evaluated the use of mHealth^a^ to promote PA^b^ mHealth devices could be used alone or in combination with other forms of interventions, such as physiotherapy. PA could be one part of the whole intervention, such as a behavior change program for weight	Studies that delivered interventions using a desktop or laptop computer Studies that used mHealth purely to monitor PA rather than deliver or guide PA
Types of outcomes	Studies that measured any outcomes on the adherence to using mHealth to promote PA	No exclusion criteria

^a^mHealth: mobile health.

^b^PA: physical activity.

### Study Selection

We used EndNote (Clarivate Analytics) to manage records identified through the electronic searches. After removing duplicate records, we screened titles and abstracts at first to identify potentially eligible studies and then screened their full texts to include eligible studies. Given that this is a scoping review, only 1 reviewer (YY) was involved in this process. However, any problem was resolved by consulting another researcher (CT or EB).

### Data Extraction

We used predefined data extraction forms and extracted the following details: (1) characteristics of the included studies: study design, study population, sample size, the description of the intervention, mHealth used, the goal of the intervention, control program, follow-up duration, and outcome measurement; and (2) factors that could influence adherence and the relationship between adherence and outcomes.

### Data Synthesis

The critical interpretive synthesis approach was used to synthesize both qualitative and quantitative data [17]. Concepts identified in the full texts of included studies were labeled. The research questions were used as a top-down coding frame. We coded text fragments that were explicitly or implicitly related to any of the following three topics: (1) adherence measurement, (2) predictors of adherence, and (3) association between adherence and intervention outcomes. Synthetic constructs (ie, concepts that explain similar themes) were developed from the codes, and relationships between the synthetic constructs were specified.

### Framework Development Methods

On the basis of the aforementioned synthetic constructs, we developed an integrative adherence framework by following the theoretical causal pathway to map the synthetic constructs of the scoping review. This framework can show the links among the determinants affecting adherence, multidimensional adherence measurements, and the association between adherence and health outcomes.

## Results

### Search Results

**Figure 1:**
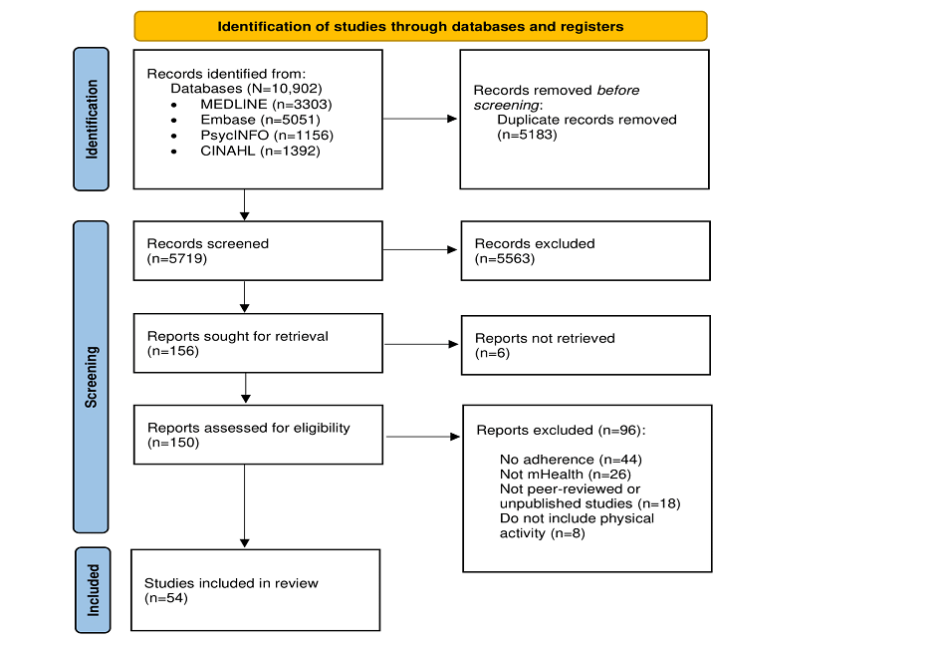
PRISMA (Preferred Reporting Items for Systematic Reviews and Meta-Analyses) flowchart of the study selection process. mHealth: mobile health.

The electronic database searches retrieved a total of 10,902 records. Title and abstract screening of these 10,902 records resulted in 150 (1.38%) requiring full-text inspection. Of these 150 papers, 54 (36%) were included in this review (Figure 1).

### Characteristics of the Included Studies

The characteristics of the included studies are summarized and reported in Textbox 1 (refer to Multimedia Appendix 3 [18-71] for full details). Most of the interventions were delivered through smartphone apps. The sample sizes of the included studies ranged from 10 to 16,948 (median 86). The average age of participants in the included studies ranged from 23.6 (SD 4.6) years to 73.2 (SD 7.3) years (median 51.7, SD 11.2).

**Summary characteristics of the included studies.**

**Types of studies**

- Randomized controlled trial design (33 studies) [18,20-22,24,25,30,31,33,34,39-47,50-53,57,58,60,61,63,65-68,71], including 9 pilot studies with small sample sizes [20,21,33,40,42,47,50,51,68]
- Pre-post quasi-experimental studies with 1 arm (10 studies) [19,23,29,37,38,49,55,56,62,69]
- Observational study design (4 studies) [26,36,48,64]
- Subgroup analyses of the intervention groups of the randomized controlled trial (2 studies) [54,59]
- Nonrandomized controlled pilot trial (3 studies) [27,28,35]
- Nonrandomized 2-arm, matched case-control trial (1 study) [60]
- Cross-sectional web-based survey (1 study) [36]

**Types of participants**

- General population (such as university students and staff or healthy adults; 10 studies) [26,35,36,39,45,48,49,57,64,70]
- People with specific characteristics (17 studies)
  Physically inactive community dwelling (4 studies) [41,53,59,65]
  People who were overweight (4 studies) [24,68,69,71]
  Older adults (4 studies) [22,23,31,37]
  Pregnant or postpartum women who were overweight (2 studies) [28,60]
  Shift workers (1 study) [47]
  Mothers (1 study) [52]
  Nurses (1 study) [55]
- People with specific diseases (27 studies)
  Diabetes or at high risk of diabetes (9 studies) [19,32,38,42,43,46,50,51,63]
  Cancer (5 studies) [20,44,56,61,62]
  Cardiac event (4 studies) [18,30,54,67]
  Musculoskeletal conditions (3 studies) [21,25,58]
  Pulmonary disease (2 studies) [27,34]
  Stroke (1 study) [33]
  Parkinson disease (1 study) [40]
  Excessive weight (in patients in primary care; 1 study) [66]
  Patients awaiting surgery (1 study) [29]

**Types of mobile health**

- Delivering interventions through a smartphone app (52 studies) [18-55,57-70]
- Using a wrist-worn activity tracker (1 study) [56]
- Using a PDA (1 study) [71]

**Functions of mobile health**

- Helping users to self-monitor and document their behavior (such as physical activity, diet, and weight; 49 studies) [18-20,22-30,32-35,37-50,53-71]
- Providing feedback, reminders, and social support (38 studies) [18,22-24,28,30,32-35,38-43,45,47,49,50,53-69,71]
- Analytic and assessment features and setting activity targets or plan (38 studies) [18,19,22-25,28,32,33,35,38-45,47,51,53-57,59-61,63-68,70]
- Providing behavior change education and instruction (30 studies) [18,21,23,25,27,28,30-34,38,40-42,44-47,50,51,54,55,58,60,61,63,66,67,69]
- Game-based function (5 studies) [35,37,39,43,51]

**Types of outcomes**

- The feasibility of the mobile health interventions
  Adherence (27 studies) [18,21,23,25-27,29-31,33-35,40,42-44,50-54,56,58,67-69,71]
  Engagement (19 studies) [20,22,28,29,32,36-39,46-49,59-62,64,65]
  Retention (10 studies) [23,28,32,42,47,48,56,59,60,64]
  Acceptability (9 studies) [20,24,28,40,53,54,60,62,68]
  Usefulness or usability (7 studies) [23,47,49,50,54,57,70]
  Satisfaction (5 studies) [33,40,56,58,68]
  Recruitment (4 studies) [28,37,56,60]
  Uptake (4 studies) [30,42,44,57]
  Completion (3 studies) [44,59,67]
  Safety (2 studies) [40,56]
  Program use (2 studies) [57,70]
  Adoption (1 study) [55]
  Implementation (1 study) [55]
  Maintenance (1 study) [55]
  Fidelity (1 study) [60]
- Change in health behavior
  Physical activity level (step count; 19 studies) [18,20,22,23,28,31,35,36,39,40,49,50,52,53,62,64-67]
  Dieting (4 studies) [28,53,62,66]
- Clinical indicators
  Change in weight or BMI (13 studies) [20,24,26,28,30,38,46,52,57,62,66,69,71]
  Physical function and walking or exercise capacity (10 studies) [19,23,25,27,31,33,34,40,50,58]
  Quality of life (9 studies) [19,31,33,34,39,40,50,65,67]
  Glycated hemoglobin levels or fasting blood glucose (5 studies) [19,30,38,51,63]
  Perceptions of treatment effectiveness (3 studies) [21,25,58]
  Blood pressure (2 studies) [30,66]
  Oxygen uptake peak (1 study) [18]
- Psychological indicators
  Physical activity motivation (4 studies) [37,42,43,51]
  Depression, anxiety stress, and mood (4 studies) [22,28,39,69]
  Self-efficacy (5 studies) [18,22,28,29,66]
  Education about heart-related health (1 study) [54]
  Adverse events (2 studies) [23,27]
  Cognitive performance (1 study) [22]
  Disease knowledge (1 study) [18]

**Follow-up**

- Range: 3 weeks to 24 months (median 12 weeks)

### Summary of Adherence Measurement Methods

We identified 31 specific adherence measurement methods used (Table 2). The top 3 most frequently used methods were manually entering self-monitored health behavior data into the device (17 studies), recording PA data (eg, step count) automatically recorded on the devices (10 studies), and recording the frequency of daily access to the app (8 studies).

These 31 measurement methods were related to 8 measurement indicators that generally reflect 4 measurement dimensions: length, breadth, depth, and interaction (Table 2). Among the 4 dimensions, the breadth dimension was the most frequently measured (35 studies). Of the 54 included studies, 31 (57%) measured adherence in only 1 dimension, 15 (28%) measured adherence in 2 dimensions, and 6 (11%) measured adherence in 3 dimensions, whereas 2 (4%) included all 4 dimensions.

**Table 2:** Adherence measurement methods.

Dimensions and measurement indicators		Specific methods reported in the included studies
**Length: the time users spend on the mHealth^a^ devices (reported by 15 studies)**		
	Device use time and frequency	Recorded the frequency of daily access to app (ie, app visit or log-in; 8 studies) [28,32,49,55,56,65,66,68] Self-reported how frequently the app was used (2 studies) [36,51] Recorded the duration of time spent on the device (2 studies) [32,60]
	Duration of use until attrition	Number of days devices were used (3 studies) [20,37,49] Time to attrition (1 study) [48] Trial retention (1 study) [68] Duration of program use (1 study) [64]
**Breadth: the proportion of mHealth functions and features used out of the total available (reported by 35 studies)**		
	Device functions used	Having physical activity data (step count and exercise) automatically recorded on the devices (10 studies) [20,35,37,40,43,59,62,64,65,70] Manually entering or uploading self-monitored health behavior data to the device (17 studies): physical activity [24,26,29,30,32,38,41,42,44,47,48,53,57,69,71], and meals, weight, and other behavioral targets [24,26,28,29,32,38,47,53,62,69] Recorded actual use and each feature (6 studies) [22,37,39,42,46,49] Recorded game played and the total duration of game played (1 study) [43]
	Completion of modules	The number of sessions attended, completed, or canceled by participants (6 studies) [23,27,48,52,54,69] Self-reported adherence to treatment or the physical activity program assessed by means of standardized questionnaires (2 studies) [31,34] Received counseling sessions (1 study) [41]
**Depth: how well the program has been used (reported by 13 studies)**		
	Meeting tasks or challenges	Self-reported adherence to the physical activity plan or target (3 studies) [31,50,58] Points won when participants achieve their daily goals (2 studies) [51,59] The duration of exercise performed vs prescribed (1 study) [54] Attendance of the planned assessment (1 study) [67]
	Behavior change (eg, physical activity level and diet habits)	Devices monitored physical activity levels such as average daily step count or physical activity time (4 studies) [18,19,48,50] Self-reported walking and sitting time (1 study) [33] and exercise time (1 study) [54] Self-reported the number of behavior targets met (2 studies) [28,45]
**Interaction: how users interact with the intervention programs (reported by 19 studies)**		
	Active interaction	Writing, or responding to, a post (6 studies) [38,55,59-61,65] Setting behavior change goals or challenges (6 studies) [22,28,45,60,63,64] Receiving and responding to SMS text messages (4 studies) [21,22,38,62] Sending digital gifts to teammates (2 studies) [59,65] The number of notifications or prompts opened and responded to (1 study) [57] Points earned when interacting with the program components (1 study) [63] Join a Facebook group (1 study) [60]
	Passive interaction	Reading articles, texts, or watching video clips through app (6 studies) [25,28,38,41,48,60] Completing telephone calls and the duration of calls (2 studies) [46,62] The number of opened notifications (1 study) [32]

^a^mHealth: mobile health.

### Factors That Affect Adherence to mHealth PA Programs

In the included studies, there are 3 factors affecting the adherence reported: user characteristics, technology-related factors, and contextual factors (Table 3). For the 5 specific user characteristics, the included studies showed inconsistent evidence on the influence of age, education status, and weight on adherence, whereas the studies by Ryan et al [59] and Guertler et al [64] consistently suggested that men had higher adherence. The study by Edney et al [39] reported that being overweight reduced adherence. The included studies consistently showed that almost all 12 technology-related factors (9 mHealth functions and 3 specific factors related to the experience of using mHealth devices) increased users’ adherence. Only 1 contextual factor was identified in this review: weekdays have higher adherence than weekends [59].

**Table 3:** Factors that affect adherence to mobile health (mHealth) physical activity interventions.

				Association with physical activity app adherence
**User characteristics**				
		Age	Inconsistent results Older age, more adherence (2 studies) [39,64] Unrelated to adherence (1 study) [51]	
		Sex	Male with higher adherence (2 studies) [59,64]	
		Weight	Inconsistent results Overweight reduced adherence (1 study) [39] Baseline weight or BMI was unrelated to adherence (1 study) [49]	
		Education	Inconsistent results Middle education category with higher adherence (1 study) [59] Higher education status increased adherence (1 study) [46]	
		Baseline physical activity	Baseline steps unrelated to adherence (1 study) [49]	
**Technology-related factors**				
	mHealth^a^ functions		Feedback on progress or motivation increased adherence (6 studies) [32,37,42,55,66,71] Networking platforms or app-specific communities increased adherence (3 studies) [36,58,66] Reminder feature increased adherence (3 studies) [57,59,66] Access to historical physical activity data increased adherence (3 studies) [32,37,47] Interpersonal contact function increased adherence (2 studies) [42,47] Tailored interventions increased adherence (2 studies) [42,47] Automation of data input increased adherence (1 study) [32] Information update increased adherence (1 study) [32] Multiple tasks decreased adherence (1 study) [55]	
	User experience		Ease of use increased adherence (2 studies) [57,66] Feeling challenged increased adherence (1 study) [37] Fun-to-use intervention increased adherence (1 study) [66]	
Contextual factors				Weekdays have higher adherence than weekends (1 study) [59]

^a^mHealth: mobile health.

### Association Between Adherence and Intervention Outcomes

Associations between adherence and intervention outcomes were reported in 13 studies. Higher adherence was reported as having associations with higher PA in 5 studies [36,39,61,65,70] or with physical function in 1 study [27], with greater weight loss in 6 studies [26,38,39,46,59,71], and with greater reductions in glycated hemoglobin levels in 2 studies [38,51]. Höchsmann et al [43] reported that participants’ adherence was positively associated with intrinsic PA motivation inventory change scores.

### Proposed Framework of the Adherence to mHealth PA Interventions

**Figure 2:**
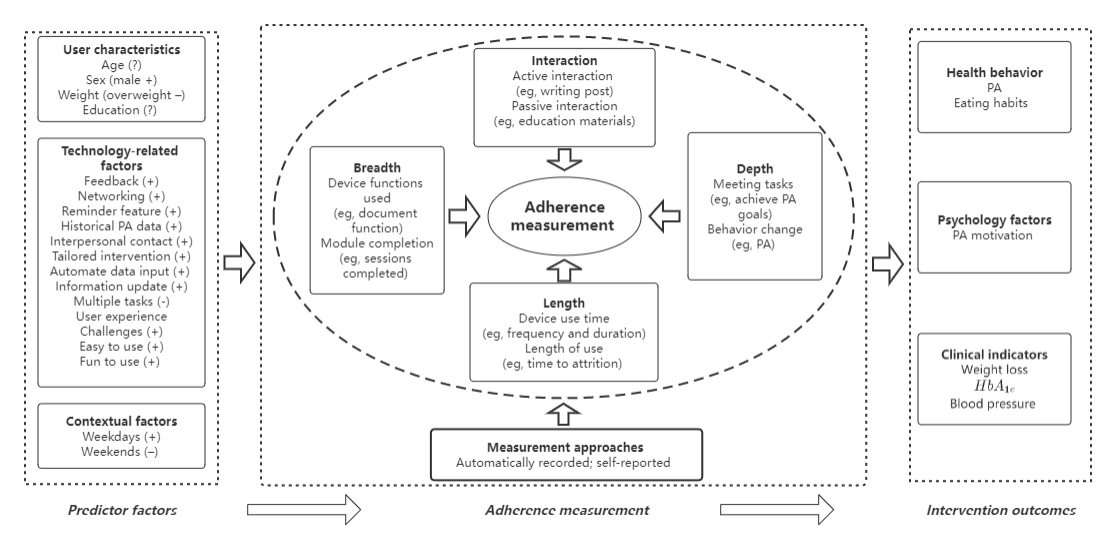
Framework of adherence to mHealth physical activity interventions. HbA_1c_: glycated hemoglobin; PA: physical activity.

The framework proposed by Perski et al [11] explicitly links potential influences on engagement and relationships between engagement and intervention effectiveness. We drew on the same structure for our adherence framework. We mapped our aforementioned review findings into a similar framework to illustrate interactions among the factors affecting adherence to mHealth PA interventions, the 4 dimensions of the adherence measurement, and intervention effectiveness (Figure 2).

Generally, most specific factors were related to aspects of user characteristics, the technology itself, and contexts that could increase users’ adherence. The adherence to mHealth could be reflected by indicators associated with 4 dimensions (length, breadth, depth, and interaction) and could be measured largely by automatically recorded data and self-reported methods. Higher adherence could improve intervention effectiveness on health behaviors as well as psychological and clinical outcomes.

## Discussion

### Principal Findings

In this scoping review, we synthesized evidence from 54 studies. The findings are as follows:

1. Users’ personal characteristics (eg, sex and education status), mHealth devices’ features, and contextual factors were reported to increase users’ adherence to mHealth PA interventions.
2. Adherence was reportedly reflected or measured in 4 dimensions and their corresponding indicators: breadth (device functions used and completion of modules), depth (meeting tasks or challenges and behavior change), length (device use time and length of use), and interaction (active or passive interaction with the program elements).
3. Higher adherence was associated with better outcomes in terms of health behaviors (eg, increasing PA), PA motivation, and clinical outcomes (eg, decrease in glycated hemoglobin levels, blood pressure, and weight).

These findings were summarized to propose an initial comprehensive adherence framework based on a theoretical causal pathway with three parts: (1) the factors that can affect users’ adherence to mHealth PA interventions, (2) multidimensional adherence measurements, and (3) the association between adherence and intervention outcomes.

### Adherence Measurement Issues

This scoping review identified 3 issues related to the measurement of adherence to mHealth PA programs in the literature. First, there is little consensus on how adherence should be defined and measured. In total, 31 specific adherence measurement methods were identified in the literature. The heterogeneity of the methods of assessing adherence makes comparison across studies difficult. Second, adherence measurement should be evidence based or theory informed; however, only the study by Adu et al [32] referred to a framework to measure adherence. Third, adherence to PA interventions is a complex definition rather than a single-dimension method [72], but many of the studies (31/54, 57%) included in this review measured adherence using only 1 dimension.

This review attempts to address the aforementioned issues through developing a new adherence conceptual framework based on the review findings. This framework, with 4 dimensions, is expected to inform the comprehensive measurement of users’ adherence to mHealth PA interventions. The four dimensions are as follows:

1. The length dimension can reflect whether the users still use the mHealth devices and how much time they spend on the devices. This dimension should be considered essential in measuring adherence and can be reflected by the time to attrition, the frequency of access to the devices, and the duration of use.
2. The breadth dimension can reflect how many device functions are involved in PA and how many modules are completed by participants. This dimension can help understand the usability of each function of an mHealth device and the engagement and involvement of users. It was the most frequent consideration in measuring adherence in the included studies. This dimension is especially worth considering when the mHealth program has multiple functions or a number of intervention modules.
3. The depth dimension can assess how well users adhere to the task of an mHealth program. It can be measured by whether participants complete the program tasks or meet the PA target. For mHealth programs included in this review, tasks were usually related to behavior changes that could be considered to reflect the depth dimension. For example, the device recorded or self-reported PA time.
4. The interaction dimension reflects how users interact with mHealth programs. In the included studies, the most frequently used methods to assess interaction included recording and counting the number of posts written or users’ responses and assessing the users’ access to educational materials.

Evidence on the topic of adherence or the related concept of engagement was included in 3 previous research studies [11,12,73]; however, all 3 differ from this review in terms of the focus of the interventions. Perski et al [11] developed a conceptual framework to highlight potential influences on engagement with a digital behavior change intervention and relationships between engagement and target behaviors. The authors’ framework specified potential direct and indirect influences on engagement but did not aim to show how the engagement could be measured. The framework we have developed in this review focuses on the topic of adherence measurement and also explores the factors that influence users’ adherence to mHealth. In addition, Perski et al [11] considered the engagement of users with a broad range of digital behavior change interventions rather than focusing on mHealth-based PA interventions. Attig et al [12] emphasized the exploration of factors that are related to the abandonment of an activity tracker rather than conceptualizing a framework for abandonment. Attig et al [12] identified less-intensive device use, less device interaction, and amount of PA achieved as important factors that affect abandonment of activity tracker use. We agree with the importance of these factors and have included them in our proposed adherence framework. Couper et al [73] analyzed data from a randomized controlled trial that aimed to evaluate the effectiveness of a web-based intervention in promoting dietary changes. They produced a 2D adherence measurement approach that consisted of breadth and depth. Breadth was defined as how widely users could access all available functions on the website, and depth was defined as how deeply users were engaged in the web-based material. Couper et al [73] considered that the *breadth-depth* engagement led to intervention outcomes: users’ retention and behavior changes. However, we consider *retention* and *behavior change* as specific aspects of length and depth, respectively. This is because both *retention* and *behavior change* are only intermediate, moderating effects in terms of mHealth use patterns, rather than determining the influence of users’ adherence on clinical outcomes resulting from PA, such as specific balance and strength outcomes. Given this, we consider our evidence-based adherence framework to be specifically relevant to mHealth-delivered PA interventions.

When choosing methods to measure adherence, researchers need to consider the following issues:

1. The characteristics of the mHealth program (eg, whether the mHealth program has multiple functions). The length and breadth dimensions are generally applicable to most mHealth programs, whereas the interaction and depth dimensions are usually applicable only to mHealth programs with relevant functions.
2. The purpose of the study. For example, if the study’s purpose is to assess the effectiveness of the mHealth intervention, users’ deep engagement with the mHealth intervention is needed, meaning that the interaction and depth dimensions should be considered.

The final issue regarding adherence measurement is how adherence data can be collected. Automatic recording and self-reporting adherence are 2 common approaches, but they both have strengths and limitations. Automatic recording can be objective but may not detect some specific types of activity (eg, stationary movement or upper or lower body movement) [74]. Self-reporting is easy to use and applicable to all types of PA, but the validity of this approach can be affected by social desirability bias [75]. The ideal way to measure adherence to mHealth PA interventions may be a combination of the objective measurement using mHealth and self-reporting.

### Factors That Influence Users’ Adherence

Some findings of this review are inconsistent with previous work: this review suggests that male sex, older age, and secondary education could be predictors of higher adherence. However, in a systematic review investigating the predictors of adherence to web-based psychological interventions, Beatty and Binnion [76] suggest that female sex and older age predict higher adherence. Another review finds that younger age is associated with higher adherence to internet interventions [77]. These disagreements may be related to the differences in the types of interventions and target populations considered.

This review found 12 technology-related factors that could affect users’ adherence, including 9 related to mHealth functions and 3 related to users’ experience. This is consistent with another systematic review in terms of technology-related factors (eg, PA tracking, PA goal setting, and customization of exercise) [78]. In the study by Attig et al [12], the reasons for abandonment included data inaccuracy, privacy concerns, discomfort, loss of motivation, and loss of tracking feasibility. This finding suggested that the mHealth devices themselves are the key to users’ adherence, and in designing mHealth, the target users could be involved to improve device functions and thus adherence.

### Adherence and Intervention Outcomes

As this review and the adherence framework demonstrates, adherence could affect intervention outcomes, including health behavior outcomes such as PA level, psychological indicators such as PA motivation, and clinical indicators such as weight loss and blood pressure. The findings suggest that further studies should explore the association between adherence and health outcomes from the aforementioned 3 perspectives.

Hawley-Hague et al [72] and Donkin et al [10] suggest that different types of clinical outcomes could be predicted using different types of adherence indicators (eg, log-ins were related to outcomes in physical health interventions, whereas module completion was most related to outcomes in psychological health interventions). Understanding how adherence influences the effectiveness of interventions could be crucial to understanding how adherence should be defined. However, this review identified only a limited number of studies that evaluate the association between adherence to mHealth and health outcomes. Further study is needed to explore in depth the association between each adherence dimension and each health outcome dimension.

### Limitations of This Review

This review includes limitations. First, given that this is a scoping review, the searches for this review were limited to publications in English; hence, evidence published in other languages could have been missed. Second, the proposed adherence framework was based on the findings of the scoping review alone; therefore, it may not include all possible factors because of the limited evidence in the literature. Further research could improve this framework by considering evidence from other sources such as expert group meetings. Third, this framework explores the adherence predictors and the association between adherence and health outcomes. However, we did not further explore how these predictors affect each adherence dimension and the associations between each adherence dimension and intervention outcomes. This is because the included studies have limited evidence regarding these issues. Further studies are needed to explore the adherence to mHealth programs by each adherence dimension.

### Conclusions

This review suggests that adherence can be measured using the dimensions of length, breadth, depth, and interaction; that users’ characteristics, technology-related factors, and contextual factors can affect adherence; and that adherence is significantly associated with outcomes in terms of health behaviors, psychology, and clinical measures. These findings inform the development of a framework, linking together the adherence predictors, comprehensive adherence assessment, and clinical effectiveness. The framework could facilitate a comprehensive measurement of adherence as well as guide mHealth device development and further studies on adherence to mHealth PA interventions. Further research is needed to validate this framework; for example, by considering evidence from other sources such as expert group meetings or using Delphi approaches.

## Appendix

Multimedia Appendix 1 PRISMA-ScR (Preferred Reporting Items for Systematic Reviews and Meta-Analyses extension for Scoping Reviews) checklist.

Multimedia Appendix 2 Example of search strategies for the MEDLINE database.

Multimedia Appendix 3 Characteristics of the included studies.

## Abbreviations

mHealth: mobile health
PA: physical activity
PRISMA-ScR: Preferred Reporting Items for Systematic Reviews and Meta-Analyses extension for Scoping Reviews
