# Supplementary material for: Measurement of Adherence to mHealth Physical Activity Interventions and Exploration of the Factors That Affect the Adherence: Scoping Review and Proposed Framework
Source: J Med Internet Res. 2022 Jun 8;24(6):e30817. doi: 10.2196/30817 (PMC9218881; doi:10.2196/30817)
Supplement: Multimedia Appendix 2 [file jmir_v24i6e30817_app2.doc]

Multimedia Appendix 2. Example of search strategies for Medline database

1. exp "Treatment Adherence and Compliance"/

2. (adher* or maintenance or maintain* or compliance or engage* or retention). ti, ab.

3. exp Telemedicine/

4. (mHealth or "mobile health" or "mobile phone" or smartphone or "smart phone" or application or APP). ti, ab.

5. (physical activit* or exercise or fitness or strength or balance). ti, ab.

6. exp Exercise/

7. 1 or 2

8. 3 or 4

9. 5 or 6

10. 7 and 8 and 9

11. limit 10 to (English language and humans)
