# Supplementary material for: Measurement of Adherence to mHealth Physical Activity Interventions and Exploration of the Factors That Affect the Adherence: Scoping Review and Proposed Framework
Source: J Med Internet Res. 2022 Jun 8;24(6):e30817. doi: 10.2196/30817 (PMC9218881; doi:10.2196/30817)
Supplement: Multimedia Appendix 3 [file jmir_v24i6e30817_app3.doc]

**Multimedia Appendix 3. Characteristics of the included studies**

| **Study ID, Country** | **Study design** | **Study population** | **Sample size (F/M);**  **mean age (SD) or**  **Range in years old** | **Descriptions of the mHealth used** | **Study**  **duration** | **Outcome measures** | **Adherence measurement methods** | **Factors that affect the adherence** | **The association between the adherence and outcomes** |
| --- | --- | --- | --- | --- | --- | --- | --- | --- | --- |
| Cai 2022  [18] China | RCT | Patients with atrial fibrillation | Intervention:  49 (18/31); 57(11)  Control: 48 (16/32); 57 (9) | Shukang app. It can provide individually pre-programmed training prescriptions, including the recommended target heart rate, single exercise records, and weekly exercise reports. With record and communicate function. | 12 weeks | Improvement in VO2 peak, adherence, physical activity, beliefs related to cardiovascular disease, and exercise self-efficacy. | Adherence: the percentage of the 12 weeks during which the patient completed 150 or more minutes of exercise. | NR | NR |
| Jiwani 2022  [19] USA | Pre-post study | Overweight  older adults with type 2 diabetes | 18 (10/8); Overall: 71.5 (5.3) | A Fitbit tracker can help to self-monitor diet and physical activity and set haviour targets. | 6 months | Frailty assessment, physical function, quality of life, and clinical laboratory measures. | Adherence to Fitbit usage for physical activity was inferred from step count data. | NR | NR |
| Allicock 2021 [20]  USA | RCT | Breast cancer survivors | 22 (22/0); Overall: 52 (9) | CHAT app. It uses an ecological momentary assessment to improve users’ physical activity and diet behaviors. | 8 weeks | Feasibility outcome: engagement and acceptability. Anthropometric measures and physical activity. | Adherence: the number of days of completed self-monitoring, and the number of valid wear days with the accelerometer. | NR | NR |
| Anan 2021  [21] Japan | RCT | Workers with neck/ shoulder pain/ stiffness and low back pain | Intervention:  48 (9/39); 41.8 (8.7)  Control: 46 (13/33); 42.4 (8.0) | AI-assisted health program, in which chatbot sent messages to users with the exercise instructions at a fixed time every day. | 12 weeks | Subjective assessment of the degree of pain, and adherence. | The number of participants who access and reply to the chatbot’s messages at least once every 3 days. | NR | NR |
| Bisson 2021  [22] USA | RCT | Sedentary Older Adults | 87 (61/26); Overall: 61.87 (7.82) | StepMATE is a fully automated app that includes behavioral supports to help people plan where and when to walk and social supports to help find others who might want to walk with them. | 4 weeks | Social engagement, exercise control, exercise self-efficacy, cognitive performance, sleep, app engagement, physical activity, mood, and energy. | The total number of SMS text messages sent to contacts, the number of routes saved, the number of scheduled events, and the number of times the *Walk Now* feature was used. | NR | NR |
| Daly 2021  [23] Australia | Pre-post pilot study | Community-dwelling older adults | 20 (10/10); Overall: 70.1 (3.1) | Physitrack is a cloud-based, digital platform that allows health professionals to assign exercises and programs to people remotely, track progress, provide feedback in real-time, and send reminders. | 8 weeks | Feasibility: retention, adherence, and adverse events. Physical activity, physical function, physical activity enjoyment, and system usability. | Adherence: the number of sessions completed, the number of exercises and sets and repetitions completed within each session was recorded within the Physitrack system. | NR | NR |
| Eisenhauer 2021 [24] USA | RCT | Overweight and obese men | 80 (0/80); Overall: 54.20 (8.59) | ‘Lost it app’: real-time self-monitoring of eating and activity, personalized reports outlining self-monitoring trends and permitting personalized goal setting, and private discussion board | 6 months | Feasibility and acceptability. Weight loss and behavior change. | Engagement and adherence: app logging for tracking weight, eating, and activity behavior. | NR | NR |
| Gruner 2021 [25] USA | RCT | Patients with knee pain | 50 (21/29); Overall: 57.1 (13.4) | Limber Health DETAb, includes assessment function, exercise video sessions, and monitor function. | 8 weeks | Pain Interference, physical function, and  Adherence. | Automatically recorded number of exercise videos watched per week. | NR | NR |
| Han 2021 [26]  low-income and OECD countries* | Retrospective cohort | Noom Coach app users | 8343 (6024/2017); Overall: 36.1 (11.7) | Noom Coach is a smartphone app for weight loss that tracks dietary intake, physical activity, and body weight. | 12 months | Adherence and  weight change. | Adherence: the frequency of exercise, diet, or the frequency of weight data entry. | NR | Weight change |
| Layton 2021  [27]  USA | Control study | Patients with severe cystic fibrosis | 11 (5/6); Overall:33 (7) | Peloton app, provides exercise videos, recorded exercise time, and corresponding heart rate. | 12 weeks | Adherence, adverse events, six-minute walk distance (6MWD), and dyspnea. | Adherence: number of sessions completed. | NR | A clinically meaningful lower decline in 6-minute walk distance |
| Napolitano 2021 [28]  USA | Controlled pilot trial | Postpartum African American/Black Women | 136 (136/0); Overall: 27.8 (5.4) | BeFAB app aims to address postpartum weight. The app includes didactic lessons delivered via a virtual coach, app-based messages, goal setting and tracking, and edutainment videos. | 12 weeks | Feasibility outcome: recruitment, retention and engagement, and  self-report acceptability.  Behavioral outcome: diet and physical activity. Psychosocial outcome: stress, coping, social support, self-efficacy. Other outcomes: weight change. | Automatically recorded: accessing the app, recording weight through the app, watching the video, setting goals; achieving activity goal, and achieving a nutritional goal. | NR | NR |
| Shelton 2021  [29] USA | Pre-post study | Patients Awaiting Abdominal Colorectal Surgery | 227 (118/109); Overall: 55.6 (15.9) | Seamless MD Digital Platform: collecting baseline health beliefs and mindsets, daily exercises, and weekly diet recall. | 4 weeks | Self-efficacy, engagement, and adherence. | Number of days users self-reported exercise  and diet using the app. | NR | NR |
| Yudi 2020  [30] Australia | RCT | Patients with acute coronary syndromes | 168 (26/142); Overall: 56 (10) | A smartphone app, with the function of exercise prescription, dynamic tracking of cardiovascular risk factors, assessment of dietary habits, heart health education, and interactive and personalized feedback and support. | 8 weeks | 6-minute walk  distance, uptake, adherence, change in fasting lipid levels, hemoglobin A1c, blood pressure, BMI, waist  circumference and smoking status. | Adherence: attending or uploading physical activity. | NR | NR |
| Taraldsen 2020 [31]  Norway, Germany, Netherlands | RCT | 61–70  Year-old community-dwelling adults | 180 (93/87);  aLiFE: 66.19 (2.32);  eLiFE: 66.43 (2.33)  Control: 66.4 (2.71) | eLiFE: Smartphone app which can deliver Lifestyle-Integrated Exercise (LiFE) instructions through video clips, pictures, and text/verbal instructions. | 12 months | Feasibility outcomes: uptake, adherence, feasibility, usability, and adverse events.  Late-life function and disability index, physical behaviour complexity metric, general health and function, medication use, neuropsychological measures, physical function, and quality of life. | Monthly self-reported adherence question.  Exercise Adherence Reporting Scale (EARS) | NR | NR |
| Adu 2020 [32]  Australia | Observational study | People with type 1 or type 2 diabetes | 50 (29/21); Overall: 49.1 (12.3) | My Care Hub, which provides support and education that facilitates positive behavioural change in diabetes management with documentation, analytics, and  education function. | 3 weeks | Retention and engagement. | App usage (extracted from the app’s database): number of active users, frequency of daily access to the app, time spent on the app, the number of notifications opened,  Data log: manually input data exercise, food activity, and weight data. | Automation of data input (+)  More analytic histories (+)  Information update (+)  Feedback on physical activities (+) | NR |
| Grau-Pellicer 2020 [33]  Spain | Pilot RCT | Stroke survivors | 41 (20/21);  Intervention: 62.96 (11.87)  Control:  68.53 (11.53) | Fitlab® Training and Fitlab® Test app: supervising and monitoring adherence to physical activity; conducting assessment and providing feedback. | 8 weeks | Primary outcome: adherence.  Secondary outcomes: walking ability, risk of falling, quality of life, and participants’ satisfaction | Community ambulation (self-report outdoors walking time).  Sedentary behaviour (self-report sitting time). | NR | NR |
| Jiménez-Reguera 2020 [34]  Spain | RCT | COPD patients | 44 (14/30);  Overall:  68.11 (6.74) | HappyAir app: providing patients useful information and advice about their illness; collecting data related to physical activity and disease; reminding users to record physical activity, medication and mood. | 12 months | Treatment adherence, quality of life, and exercise capacity. | Adherence to the programme was assessed by the respiratory physiotherapy adherence self-report questionnaire.  Adherence to physical activity was assessed by a therapeutic compliance questionnaire. | NR | NR |
| Liew 2020 [35]  Singapore | Controlled pilot trial | Staff and students from a public university | 40 (20/20);  Overall:  24 (23 to 30) | A smartphone app, and a Web-based data management unit with the following features: team formation, setting challenges, gamified features, communication features, and providing feedback. | 6 weeks | Adherence, step count, and floor count. | The number of valid participants: who contributed valid step data. | NR | NR |
| Petersen 2020 [36]  Australia | Cross-Sectional Study | Adults who currently use and not use PA app | 1432 (1256/176);  Overall: 34.1 (18 to 83) | Any commercial physical activity apps. | -- | Self-reported regular structured physical activity, app usage, and engagement. | Self-report engagement with the features of the app-specific community on a 6-point Likert scale ranging from 0 (never) to 5 (very often). | App-specific communities (+)  Social networking platforms (+) | The dose of app usage was significantly associated with PA.  Sharing physical activity posts predicted engagement in PA. |
| Tabak 2020 [37]  Netherlands | Pre-post study | Older adults (65–75 years) | 20 (10/10);  Overall: 71.0 (5.0) | WordFit, is a game-based coaching app using step count data (FitBit) for the crossword-inspired game. | 4 weeks | Engagement and motivation.  Semi-structured interviews (user experience). | The number of days when devices were used (measured by recording at least 100 steps counts).  Recording that app has been opened.  Recording at least one word has been placed on the board within the game. | Document step data (+) Feedback on performance (+)  Challenge features (in-game challenges, and challenges through emergent gameplay) (+) | NR |
| Toro-Ramos 2020 [38]  USA | RCT | People with Prediabetes | 202 (144/58);  Intervention: 55.7 (13.6)  Control: 57.5 (12. 5) | Noom Coach app, with features of messaging, daily challenges, education articles; logging food, steps, and exercise, and then getting automated feedback | 12 months | Change in weight and HbA1c levels, and engagement. | Recording weekly numbers of logged meals, logged weigh-ins, logged steps, articles read, posts in the group, and messages to the coach. | NR | Program engagement behaviours predicted changes in weight and HbA1c levels at 6 and 12 months |
| Edney 2019 [39]  Australia | RCT | Facebook users who had <150 moderate-to-vigorous PA each week | 301 (222/79);  Overall: 41.8 (11.9) | Active Team app: designed to encourage adults  to increase their physical activity by taking 10,000 steps per day for 100 days (log and track steps, simple gamification, communication, and reminder) | 100 days | Primary outcome: change in objective physical activity.  Secondary outcomes: self-reported physical activity, quality of life, depression, anxiety and stress, well-being, and engagement. | Automatically uploaded real-time log of each user’s interactions with the app. | Overweight (-)  Older users (+) | Superusers were completing 28.2 more minutes of objectively measured physical activity than regular users. |
| Ellis 2019 [40] USA | Pilot RCT | People with Parkinson’s Disease | 51 (23/28);  Overall: 64.1 (9.5) | An app with elements of remote  monitoring, more accessible communication, and more frequent program adaptation by a physical therapist. | 12 months | Objectively measured physical activity, health-related QoL, walking capacity, adherence, safety, and acceptability. | Daily records of steps and exercises performed by the app. | NR | NR |
| Fukuoka 2019 [41]  USA | RCT | Physically Inactive Women | 210 (210/0);  Overall: 52.4 (11.0) | The mPED app can increase physical activity with two main functions: (1) a daily message or video clip and (2) a daily physical activity diary. | 6 months | Primary outcome: daily total steps.  Secondary outcome: time spent in moderate to vigorous physical activity. | Receiving in-person counselling sessions.  Adherence to watching daily messages or video clips and completing daily physical activity diary via the study app. | NR | NR |
| Griauzde 2019 [42]  USA | Pilot RCT | Adults with Prediabetes | 69 (44/25);  Overall: 51.7 (11.2) | A smartphone app that can chart the daily health-related habits and behaviours and chart their alignment with personal core values. | 12 weeks | Primary outcome: intervention uptake, retention, and adherence.  Secondary outcome: autonomous motivation | The number of days that users entered data into the app. Recorded the number of total days that the devices were used during the intervention period. | Feature of encouraged self-reflection (+)  Consider personal circumstances (+)  Interpersonal contact (+) | NR |
| Höchsmann 2019 [43] Switzerland | RCT | Overweight type 2 diabetes patients | 36 (17/19);  Intervention:56.0 (5.0);  Control: 58.0 (6.0) | Mission Schweinehund, which is a smartphone game included rewards for successful physical activity behavior, goal setting, action planning, feedback on performance, and prompts and cues were incorporated into the game mechanics to support  sustained changes in PA behavior. | 24 weeks | Intrinsic Motivation Inventory (IMI),  physical activity adherence | Recorded usage data from the app: daily physical activity (steps per day), completed and cancelled in-game workouts, and patterns and total duration of game use. | NR | A significant positive association between total in-game training (min) and change in IMI total score |
| Lee 2019 [44] Republic of Korea | RCT | Prostate cancer patients undergoing surgery or androgen deprivation therapy | 98 (0/98);  Intervention: 69.06 (7.21);  Control: 69.82 (7.73) | An app provided a weekly goal and conclusion, video clips of prescribed resistance, and stretching exercises/ nutrition information. | 12 weeks | Uptake, adherence, and completion of exercise intervention. | Adherence was defined as uploading or recording at least three sets of data (resistance exercise they performed) per week for a minimum of 8 weeks during a period of 12 weeks. | NR | NR |
| Ni Mhurchu 2019 [45]  New Zealand | Cluster RCT | Adults in the māori and Pasifika communities | 1451 (1012/439);  Intervention: 38·9 (13·4);  Control: 36·3 (12·1) | OL@-OR@ app provided information on healthy eating and physical activity, culturally relevant information, and links to local activities and services. | 12 weeks | The primary and secondary outcomes: self-reported adherence to health-related behavioural guidelines at 12 weeks and 4 weeks, respectively. | Self-reported composite health behaviour score  (the number of health guidelines met).  Programme engagement: setting at least one behaviour change goal within the app or website. | NR | NR |
| Muralidharan 2019 [46] India | RCT | Smartphone users who are at high risk for type 2 diabetes | 561 (241/320);  Intervention: 37.8 (9.2);  Control:37.8 (9.6) | The app (mDiab), which enabled tracking participants’ weight, physical activity, and diet along with 12 weekly video lessons on T2D prevention. | 12 weeks | Weight loss, and engagement. | Engagement was classified into three categories: (1) participants who attended coach calls only, (2) participants who used the App only (no detailed description), and (3) participants who did both. | Individuals with some form of education showed (+) | Individuals who viewed videos and attended coach calls had more weight loss |
| Oftedal 2019 [47]  Australia | Pilot RCT | Shift-workers | 40 (21/19);  Overall: 35.7 (9.5) | A smartphone app where users can set goals for and self-monitored physical activity, diet quality, and sleep/ and updated these goals. A traffic light feature dashboard to provide dynamic feedback on their performance. | 4 weeks | Recruitment, engagement, attrition, usefulness ratings, system usability scale, qualitative interviews, and treatment eﬀect. | Participants entered data (physical activity, diet, sleep) into the app. | Tailored intervention (+) Accessible information (+)  Interactive features (+) | NR |
| Rayward 2019 [48]  Australia | Observation studies | New users of the 10,000 Steps program | 1242 (996/245);  Overall: 44.6 (12.7) | The 10,000 Steps programme smartphone app, enables an accumulation and self-monitoring of physical activity. | 6 weeks | Social media campaign costs, engagement, and time to non-usage attrition. | The average number of sessions attendance, the average number of pages viewed per session, average number of step entries, average daily step count, and time to attrition. | NR | NR |
| Tong 2019 [49]  Australia | Pre-post study | Healthy adults | 55(28/27);  Overall: 23.6 (4.6) | A smartphone app that enables users to compare step counts with others; Social forums and private messages allow participants to interact and provide social support. | 6 months | Primary outcome: change in daily step count.  Secondary outcome: engagement with the intervention, and system usability. | Engagement: length of usage (the mean number of days of usage) and frequency of usage (recorded the number of times participants used the app and each feature in a day). | No statistically significant differences in any characteristics between frequent and nonfrequent app users: baseline weight; baseline BMI; baseline steps/day | NR |
| Valentiner 2019 [50]  Denmark | Pilot RCT | Patients with type 2diabetes with a Body Mass Index of 18 and 40 kg/m | 37 (24/13);  Overall: 65.9 (6.8) | InterWalk App includes audio guidance, an individualised fitness test, an IWT history, logging of accelerometer, and Global Positioning Systemdata during the IWT sessions. | 12 weeks | Primary outcome: adherence.  Secondary outcome: usability, satisfaction, physical activity, quality of life, and aerobic capacity. | Recorded total accumulated minutes of interval walking using data from the InterWalk App.  Self-reported adherence to interval walking training based on two questions. | NR | NR |
| Dugas 2018 [51]  USA | Pilot RCT | Older veterans with poorly controlled Type 2 diabetes | 27 (3/24);  Overall: 67.6 (5.9) | A gamified mHealth tool (DiaSocial) aimed at encouraging tracking of glucose control, exercise, nutrition, and medication adherence. | 13 weeks | Regulatory mode,  adherence, and changes in HbA1C. | Adherence was assessed by the allocated daily points (achieving their daily goals related to reporting and reaching target levels of glucose, exercise, nutrition, and medication adherence). | Age was unrelated to total adherence. | Greater adherence was associated with greater reductions in glycated haemoglobin (HbA1c) levels |
| Mascarenhas 2018 [52]  USA | RCT | Healthy mothers who have at least 1 child under the age of 12 years | 64 (64/0);  Intervention: 36.8 (6.5);  Control: 37.3 (4.0) | A list of recommended mobile exercise apps (eg, Nike+, Sworkit) of their choice. | 8 weeks | Primary outcome: self-reported physical activity.  Secondary outcome: self-reported weight, and  adherence. | Adherence: session attendance per week throughout their 8-week participation. | NR | NR |
| Spring 2018 [53]  USA | RCT | Adults with low fruit and vegetable, low PA, and high sedentary time | 212 (162/50);  Overall: 40.8 (11.9) | Make Better Choices 2 app with the function of receiving behavioural feedback, and reporting sedentary leisure screen time. | 9 months | Primary outcome: composite diet and activity improvement score.  Secondary outcome: treatment fidelity, receipt, and adherence. | Self-monitoring adherence: the proportion of days that participants used the app to record targeted behaviors. | NR | NR |
| Salvi 2018 [54]  Spain, Germany, and the United Kingdom | Subgroup analysis of an RCT | Patients who had suﬀered a cardiac event | 132 (15/117);  Overall: 59.0 (14.0) | The GEx system (mobile, patient, and professional station) can provide exercise monitoring, guidance, motivational feedback, and educational content. | 6 months | Acceptance, perceived usefulness, education about heart-related health, exercise adherence, system reliability. | Percentage of started and cancelled sessions, and minutes of exercise performed versus prescribed (device recorded).  Exercise time (in minutes) and effort (self-reported) compared to the total number of minutes prescribed. | NR | NR |
| Torquati 2018 [55]  Australia | Pre-post pilot study | Nurses | 47 (41/6);  Overall: 41.4 (12.1) | The app offered prompts and support for the participant to pre-set dietary and PA goals | 9 months | Primary outcome: changes in physical activity behaviour,  adoption, implementation, maintenance, and qualitative interview. | The frequency of use of intervention tools (pedometer, Facebook group, smartphone app). Measured by recording the number, date, type, and views of posts delivered through Facebook. | Multiple tasks (-) | NR |
| Trinh 2018 [56]  Canada | A pre-post study | Men who were diagnosed with prostate cancer | 46 (0/46);  Overall: 73.2 (7.3) | Jawbone UP 24: a wrist-worn tracker to assess activity patterns and provide sensory alerts after prolonged sitting, set update target according to performance. | 12 weeks | Primary outcomes: recruitment, completion, attrition, adherence, safety, and satisfaction.  Secondary outcome: physical activity and sedentary behavior. | Intervention adherence was defined as the number of logins > 3 visits each week (tracked through website analytics). | NR | NR |
| Levin 2017 [57]  USA | A pilot RCT | Community adults | 23 (13/10);  Overall: 26.9 (8.7) | A novel ACT-based app for improving health behaviours based on the matrix approach. | 2 weeks | Primary outcome: weight control.  Secondary outcome: system usability and program usage. | Recorded the number and timing of prompts received and responses to each prompt. | Simple and easy to use (+)  Remind participants of their goals and the function of their actions (+) |  |
| Lambert 2017 [58]  Australia | RCT | People with musculoskeletal conditions | 80 (28/52);  Overall: 48.0 (17.0) | A smartphone app that can deliver exercises prescribed by each patient’s treating physiotherapist. | 4 weeks | Primary outcome: self-reported adherence.  Secondary outcome: functional performance, disability, satisfaction, perceptions of treatment effectiveness, and different aspects of adherence. | Self-reported exercise adherence on a numerical scale ranging from 0 = ‘never  performed my exercises’ to 10 = ‘always performed my exercises’. | NR | NR |
| Ryan 2017 [59]  Australia | Subgroup analysis of the intervention arm of an RCT | Participants between 18 and 65 years old, performing less than 150 min of MVPA per week | 51 (37/14);  Overall: 35 (12.5) | Facebook app with function including recording and tracking step counts; social features such as virtual gifts, a team discussion board, and a team tally board that ranks team members based on step counts. | 8 weeks | Engagement, compliance, and retention with Active Team. | Engagement: number of step log occasions, wall posts made to the team discussion board, virtual gifts sent to teammates, and gamification score. | Middle education category (+), males (+)  Customized, periodic push reminders feature (+)  Engagement peaked on Wednesdays | NR |
| Willcox 2017 [60]  Australia | RCT | Overweight pregnant women | 91 (91/0);  Overall: 32.5 (3.4) | A smartphone app can provide tailored text messages, information websites, video messages, and chat room interaction. | 36 weeks | Feasibility: recruitment, retention, fidelity, engagement, and acceptability.  Changes in gestational weight gain, and self-reported intake and physical activity. | Contact with the programme elements (set target), text programme (receive and review text and reply text and report reading these text), join the Facebook group, or submit a post. | NR | NR |
| Valle 2017 [61]  USA | RCT | Young adult cancer survivors | 86 (78/8);  Overall: 31.7 (6.2) | Facebook-based physical activity intervention with guidance on behavioural strategies (e.g., self-monitoring, enlisting social support) | 12 weeks | Facebook engagement. | Number of responses and posts made by users.  Self-reported: how often they engaged with Facebook, with response options ranging from 1 = less often or never to 6 = several times a day. | NR | Responses posted on Facebook were significantly associated with physical activity at 12 weeks. |
| Quintiliani 2016 [62]  USA | Pre-post study | Overweight breast cancer survivors | 10 (10/0);  Overall: 59.0 (6.0) | A smartphone app with functions of self-monitoring nutrition behaviour, weight, and physical activity. Technology-assisted phone counselling from a behavioural health counsellor. | 10 weeks | Feasibility outcome: engagement and acceptability.  Evaluation outcomes: weight, diet, and physical activity. | Number of calls completed, duration of calls, number of responses to text messages, and valid days of wearing the wristband pedometer and recording a weight on the scale. | NR | NR |
| Block 2015 [63]  USA | RCT | Prediabetes | 339 (106/233);  Overall: 55.0 (8.9) | An Android and iPhone app permits users to select weekly goals, report on progress and set mobile phone reminders. | 6 months | Adherence, changes in fasting glucose and HbA1c. | The points each participant earned through interacting with the programme components each week. Assessing participants’ weekly goal-setting behaviours. | NR | NR |
| Guertler 2015 [64]  Australia | Observational study | The 10,000 steps program users aged at least 18 years | 16948  (11841/5107);  Overall: 41.8 (12.1) | A smartphone app allows users to enter their daily physical activity, join and view the progress of challenges. | 3 months | Engagement, non-usage attrition, physical activity, and steps count per day. | The duration of programme used, the number of individual challenges initiated, the number of workplace challenges initiated, and the total number of days PA was recorded in the step log. | Older age (+), male (+), and being non-Australian (+)  Challenges features (individual and workplace challenges) (+) | NR |
| Maher 2015 [65]  Australia | Cluster RCT | Inactive Facebook users | 110 (82/28);  Overall: 35.6 (12.4) | A Facebook-based physical activity intervention that enables friendly rivalry within friendship groups, offer peer encouragement and support. | 50 day | Primary outcome: Self-reported weekly physical activity time.  Secondary outcome: overall QoL, mental health QoL, and engagement. | Adherence was assessed via usage statistics: the number of visits to the app, participants’ step-logging patterns, number of virtual gifts sent, and number of posts on the message walls. | NR | “High dose” participants increased their moderate-to-vigorous physical activity (MVPA) significantly more than “low dose” participants. |
| Laing 2014 [66]  USA | RCT | Overweight primary care patients | 212 (154/58);  Overall: 43.3 (14.3) | A calorie counting app that provides a database of more than 3 million foods and an easy-to-use interface for logging food and exercise. | 6 months | Primary outcome: change of weight.  Secondary outcome: systolic blood pressure, self-reported behavioural change, exercise, dieting, and self-efficacy. | Frequency of app logins over time (each time a participant opened the app counted as a login). | Easy to use (+); receiving feedback (+); fun to use (+), reminder feature (+), the social networking feature (+).  Logins decreased sharply after the first month | NR |
| Varnfield 2014 [67]  Australia | RCT | Myocardial infarction patients | 120 (82/38);  Intervention: 55.7 (10.4);  Control: 55.5 (9.6) | An app that can monitor health and exercise, deliver motivational and educational materials. A web portal for mentors to provide weekly consultations. | 6 months | Primary outcome: uptake, adherence, programme completion.  Secondary outcome: physical activity, nutrition, psychosocial functioning,  biomedical risk factors, and health-related quality of life. | Uploading of 4 weeks’ exercise data.  Attending 6-week assessment. | NR | NR |
| Carter 2013 [68]  UK | Pilot RCT | Overweight volunteer | 128 (99/29);  Overall: 42.0 (9.0) | My Meal Mate (MMM) app with the functions of goal setting, self-monitoring of diet and activity, and providing feedback via weekly text messages. | 6 months | Adherence, frequency of use, acceptability, satisfaction, change in anthropometric measures. | Adherence to the trial: trial retention. Adherence to the interventions: frequency of use | NR | NR |
| Thomas 2013 [69]  USA | Pilot pre-post study | Overweight and obese men and women | 20 (19/1);  Overall: 53.0 (1.9) | An app with the functions of self-monitoring, feedback (automated and human), and brief videos for education and skills training. | 24 weeks | Changes in weight,  technology anxiety scale, and adherence. | Attendance at treatment sessions and number of days adherent to self-monitoring.  Recorded the average day’s physical activity was reported, and physical activity time.  Recorded the number of logins, videos viewed, and the use frequency of the personalized behavioural monitoring feature. | NR | Adherence to the self-monitoring protocol was correlated with weight loss at 12 weeks. The number of video lessons viewed was not associated with weight loss. |
| Kirwan 2012 [70]  Canada | A 2-arm matched case-control trial | Existing members of the 10,000 Steps program | 200 (96/104);  Overall: 39.3 (12.8) | The iStepLog app that allowed participants to record their daily physical activity on their mobile device and synchronise this information with their online Step Log. | 3 months | App usage, the usability and usefulness questionnaire. | Total number of steps logged, the total number of days steps were logged, and time spent using the app on each occasion. | NR | App usage increased the likelihood to log greater than 10,000 steps on each entry |
| Burke 2011 [71]  USA | RCT | Overweight/obese adults | 210  (164/:46);  Overall: 46.8 (9.0) | PDA devices with functions of monitoring diet, providing daily feedback through message. | 24 months | Weight change, and  adherence. | The percentage of days that participants recorded calories consuming in their diaries. If a diary was not submitted, nonadherence was assumed. | NR | Weight loss was greater for those who were adherent >60% versus < 30% of the time (p < 0.001) |

Note: ‘+’= positive association with adherence; ‘-’ = negative association with adherence, NR = Not report, *OECD countries = Organization for Economic Co-operation and Development (OECD) countries.
